# Spatiotemporal Trends in the Incidence of Gastrointestinal Neoplasms in Wuwei City of Northwestern China From 1995 to 2016: A Hospital-Based Retrospective Observational Study

**DOI:** 10.3389/fonc.2021.712857

**Published:** 2021-09-03

**Authors:** Kun Liu, Shuxuan Song, Ting Fu, Yiwen Liu, Hui Zhang, Min Yan, Zhen He, Weilu Zhang, Haixia Su, Zhao Li, Zhaohua Ji, Zhongjun Shao

**Affiliations:** ^1^Department of Epidemiology, Ministry of Education Key Lab of Hazard Assessment and Control in Special Operational Environment, School of Public Health, Air Force Medical University, Xi’an, China; ^2^Department of Immunization Program, Wuwei Municipal Center for Disease Control and Prevention, Wuwei, China; ^3^Department of Prevention of Infectious Diseases, Xi’an Center for Disease Control and Prevention, Xi’an, China; ^4^Health Commission of Wuwei, Wuwei, China

**Keywords:** gastrointestinal neoplasms, epidemiology, temporal and spatial distribution, joinpoint trend analysis, public health

## Abstract

**Objective:**

To determine the characteristics and spatiotemporal distribution of major gastrointestinal (GI) neoplasms in inpatients from 1995 to 2016 in Wuwei city, northwestern China.

**Method:**

Data from all paper and electronic medical records entered between 1995 and 2016 at 12 major public hospitals in Wuwei city were retrospectively collected. Patients with GI neoplasms were identified and classified according to the International Classification of Diseases (ICD)-10. Trends in the incidence of major GI neoplasms were expressed as an annual percentage change (APC), and the Z test was used to assess the time fluctuation trends. Age-standardized incidence rates (ASIRs) were also calculated and the corresponding APC was estimated by the Joinpoint software for long-term trend analysis. Thematic maps of annual incidence at the township level were produced.

**Results:**

Among the 19,137 new inpatients identified with GI neoplasms in Wuwei, gastric cancer was the leading cause of morbidity, followed by cancers of the esophagus, colorectum, gastric cardia, liver, and pancreas with ASIRs of 21.8, 11.0, 5.8, 5.7, 4.4, and 1.7 per 100,000 person-years, respectively. Overall, there was a steady increase in the ASIR for all GI neoplasms, and male cases were 2.1 times more frequent than female cases. The ASIR significantly increased by 12.2% per year from 1995 to 2009 for all GI neoplasms, and the increase rates ranged 9.4%-16.7% per year for the individual GI neoplasm. Despite an increase by 1.4% per year from 2009 to 2016, the ASIR decreased for esophageal and gastric cardia cancers by 4.6% and 17.3% per year, respectively. The annual incidence of all GI neoplasms showed significantly differential geographic distributions among different townships of the city during the study period.

## Introduction

As the most common group of cancers globally, gastrointestinal (GI) neoplasms are responsible for considerable morbidity and mortality (1). According to GLOBOCAN 2020, colorectal cancer (with around 1,149,000 new cases every year) is the most common GI cancer worldwide, followed by gastric cancer (about 1,089,000 every year), liver cancer (about 905,000 every year), and esophageal cancer (about 604,000 every year) (2). Besides lung cancer and female breast cancer, GI neoplasms, such as colorectal, gastric, hepatobiliary, pancreatic, and esophageal cancer, are the main burden in China (3). Gastric cancer was the leading cause of cancer mortality in China from 2010 to 2014, followed by esophageal and colorectal cancers (4). Although various studies have reported the annual mortality rates of common GI cancers (4–6), the annual linear trends of GI neoplasm rates among different age and sex groups have not been comprehensively assessed in China.

According to the 2015 Cancer Statistics, the population in northwestern China has the highest incidence of gastric cancer and the second highest incidence of esophageal cancer (5). Wuwei, a city located in Gansu Province of northwestern China, has a high prevalence of GI neoplasms, especially those in the upper GI tract in China (7, 8). With the highest incidence of gastric cancer in China, there were about 11,000 cases with GI neoplasms recorded in Wuwei during 2006 to 2011, with an annual incidence of 190.3 per 100,000 person-years, which is much higher than the national average (9). However, the overall or age-sex specific trends in the incidence of GI neoplasms in Wuwei have not been systematically investigated.

Our study aimed to illustrate the trends in the incidence of major GI neoplasms and compare the incidence rates among different age and sex groups in Wuwei City. The epidemiological characteristics of six major GI cancers were assessed using longitudinal data from 12 public hospitals from 1995 to 2016 in Wuwei. The information not only serves as a valuable reference for policy-makers and funding agencies, but also allows the comparison of GI neoplasms burden in Asia to that of the Western counterparts and highlights important questions for future research.

## Materials and Methods

### Study Area

Wuwei (north latitude 36°29’–39°27’, longitude 101°49’–104°16’) is situated at the Eastern region of the Hexi Corridor of Gansu Province, northwestern China. It consists of one district (Liangzhou) and three counties (namely, Minqin, Gulang, and Tianzhu) with a total of 102 townships, and covers a total area of 32,347 km^2^. According to the population census in 2016, there are 1,819,800 permanent resident population in Wuwei (6, 9).

### Data Source and Collection

Data were collected from all paper and electronic medical records of 12 major public hospitals which serve 90% of the population and are responsible for 81% of the hospital beds in Wuwei from 1995 to 2016 (**Figure S1**). Patients who were admitted into the 12 public hospitals and histologically confirmed with GI neoplasms were identified and classified by the International classification of Diseases Tenth revision (ICD-10) (10). Patient information such as age, sex, date of diagnosis, length of hospital stay, medical expenses, and residential address were collected. Length of hospital stay was determined from the date of hospital admission and discharge home, and medical expenses included the cost of Western medicine and Chinese medicine, bed fee, examination fee, laboratory fee, medical treatment fee, operation fee, nursing fee, and other costs. Each case was geo-referenced to a digital map of Wuwei according to the patients’ residential addresses. Demographic data were obtained from the local Statistical or Public Security Bureaus. The studies involving human participants were reviewed and approved by Xi ‘an Center for Disease Control and Prevention Ethics Review Committee (XA20170654). All study-related information was analyzed anonymously.

### Statistical Analysis

An inpatient who suffered from one GI neoplasm was counted only once for the particular GI neoplasm in the year of diagnosis, and a patient who suffered from more than one GI neoplasms was counted once for the overall GI neoplasms, and only once for the particular GI neoplasms in the years of diagnosis. Because of the special pathologic features, treatment, and prognosis of gastric cardia cancer, the data of gastric cancer (ICD-10 code: C16) did not include the data of gastric cardia cancer (ICD-10 code: C16.001). According to the medical cognition level, treatment level, and economic level in different periods, we divided the study period into four periods: 1995–2000, 2001–2005, 2006–2010, and 2011–2016. Descriptive and spatiotemporal analysis were performed after database construction. Temporal trends in incidence rates from 1995 to 2016 were examined by fitting joinpoint models to log-transformed age-standardized rates (per 100,000 population) according to the world standard population (5, 11). To reduce the possibility of reporting spurious changes in trends over the period, all models were restricted to a maximum of two joinpoints (three line segments). Trends in the incidence of major GI neoplasms were expressed as an annual percentage change (APC), and the Z test was used to determine the trends (i.e., increase, no change, and decrease) by assessing whether the APC was statistically different from 0. The increased or decreased trend was defined when the APC slope of the trend was statistically significant (P < 0.05). The results of the APCs for all GI cancers combined and the six most common GI cancers stratified by sex were presented. Age-standardized incidence rates (ASIRs) were calculated and the corresponding APCs were estimated by the Joinpoint software for long-term trend analysis (12, 13). In addition, thematic maps of annual incidence were produced in the ArcGIS version 10.1 software (ESRI Inc., Redlands, CA, USA) to characterize the temporal and spatial distribution of the major GI neoplasms at the township level of the city during the study period.

## Results

### Incidence Overview

**Figure 1:**
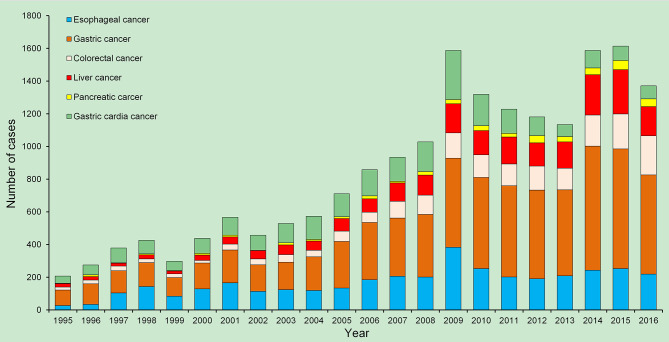
Temporal trends in the numbers of the major gastrointestinal (GI) cancer cases in Wuwei, northwestern China, 1995–2016, China.

A total of 19,137 inpatients with GI neoplasms were recruited and the numbers of patients diagnosed with each major cancer over the years are shown in **Figure 1**. The epidemiologic features of the major GI neoplasms are summarized in **Table 1**. Gastric cancer was the leading cause of morbidity, followed by cancers of esophagus, colorectum, gastric cardia, liver, and pancreas and hepatobiliary system with ASIRs of 21.8, 11.0, 5.8, 5.7, 4.4, and 1.7 per 100,000 person-years, respectively. There was a steady rise in the crude incidence rates (CIRs) and ASIRs for all GI neoplasms, and the numbers of male cases were 2.1 times higher than female cases, which presented different trends (**Tables 1**–**3**). The ASIR for all GI neoplasms increased significantly from 1995 to 2009 by 12.2% per year; the ASIRs increased by 9.4% per year for esophageal cancer from 1995 to 2009, 10.5% per year for gastric cancer from 1995 to 2014, 11.0% per year for colorectal cancer from 1995 to 2009, 11.2% per year for pancreatic and hepatobiliary cancers from 1995 to 2016, 16.7% per year for liver cancer from 1995 to 2009, and 9.9% per year for gastric cardia cancer from 1995 to 2009. However, the ASIR for gastric cardia cancers decreased by 17.3% per year from 2009 to 2016 (**Table 3**).

**Table 1:** Epidemiologic features of GI neoplasms in Wuwei, northwestern China, 1995–2016.

Variable	Esophagus cancer	Gastric cancer	Colorectal cancer	Liver cancer	Pancreatic cancer	Gastric cardia cancer
ICD-10	C15	C16	C18–C21	C22	C23–C24	C16
No. patients*	3746	7818	2017	2271	641	2644
Male/female	1.23	3.56	1.41	2.84	1.7	1.62
Age, mean ± SD	62.5 ± 9.18	59.6 ± 11.1	59.6 ± 10.8	58.7 ± 12.3	62.6 ± 16.5	60.3 ± 12.6
Length of hospital stay (day), mean ± SD	22.1 ± 18.1	13.8 ± 14.3	16.2 ± 13.7	13.8 ± 15.8	12.5 ± 13.6	16.0 ± 14.5
Medical expense (¥), median (range)	8,612.9 (3.8, 237,517)	4,000.1 (19.6, 218,851.1)	5,323.7 (57.0, 103,383.0)	3,819.9 (113.7, 181,358.9)	7,209.9 (94.9, 52,679.3)	6,184.7 (12.0, 58,294.8)
No. pathologic diagnosis (%)	2,366 (63.3%)	4,485 (57.4%)	909 (46.2%)	385 (17.0%)	207 (32.9%)	1,528 (58.8%)
No. operation (%)	960 (25.6%)	3,363(43.0%)	805 (40.9%)	391 (20.2%)	213 (33.9)	1,311 (50.4%)

GI, gastrointestinal; ICD-10, International Classification of Diseases 10; SD, standard deviation.

**Table 2:** Annual crude incidence rates and age-specific and standardized incidence rates (1/100,000 person-years) for GI neoplasms in Wuwei, northwestern China, 1995–2016.

Year	Esophagus cancer		Gastric cancer		Colorectal cancer		Liver cancer		Pancreatic cancer		Gastric cardia cancer		All GI cancers	
	CIR	ASIR	CIR	ASIR	CIR	ASIR	CIR	ASIR	CIR	ASIR	CIR	ASIR	CIR	ASIR
1995	1.4	1.9	5.0	6.3	1.0	1.3	1.1	1.4	0.1	0.1	2.3	2.9	11.1	14.1
1996	1.8	2.3	6.5	8.1	1.2	1.6	1.1	1.3	0.0	0.0	3.2	4.1	14.6	18.5
1997	5.5	7.1	7.2	9.2	1.5	1.8	1.0	1.2	0.3	0.4	4.7	6.2	20.4	26.4
1998	7.4	9.7	7.7	9.6	1.2	1.5	1.3	1.5	0.3	0.4	4.4	5.8	22.9	29.1
1999	4.4	5.8	6.1	7.7	1.2	1.5	0.9	1.3	0.2	0.3	3.0	4.0	16.2	21.3
2000	6.8	8.8	8.3	10.6	0.8	1.1	1.7	2.1	0.2	0.2	4.9	6.6	23.7	30.7
2001	8.8	11.7	10.5	13.2	1.9	2.3	2.3	3.2	0.4	0.6	5.8	7.6	30.8	40.1
2002	5.9	8.0	8.5	11.1	1.9	2.3	2.6	3.7	0.2	0.2	4.9	6.7	24.6	32.6
2003	6.5	8.9	8.8	11.3	2.4	2.8	3.1	4.0	0.4	0.5	6.2	8.1	28.5	37.2
2004	6.3	8.4	10.9	14.0	2.1	2.7	3.0	3.7	0.5	0.7	7.3	9.8	30.9	40.4
2005	7.1	9.7	14.8	19.7	3.3	4.4	4.1	5.2	0.9	1.3	7.4	10.3	38.8	52.5
2006	9.7	13.1	17.9	23.8	3.3	4.7	4.3	6.0	0.7	0.9	8.3	11.0	45.7	61.2
2007	10.7	13.7	18.5	23.6	5.3	7.1	5.8	7.5	0.9	1.1	7.6	9.7	50.1	64.4
2008	10.4	13.2	19.8	24.7	6.1	7.8	6.3	8.1	1.4	1.7	9.5	11.9	55.8	70.3
2009	19.8	24.3	27.7	33.7	8.0	9.8	8.2	9.9	1.8	2.1	15.2	18.4	83.6	102.1
2010	13.0	16.0	28.7	33.5	7.1	8.3	7.4	8.9	1.6	1.9	9.8	11.8	70.8	84.3
2011	11.0	12.9	30.3	34.9	7.3	9.2	9.1	11.0	2.8	3.8	8.1	9.4	71.0	84.1
2012	10.4	11.9	29.4	33.0	8.0	9.1	7.7	9.0	3.2	3.7	6.3	7.3	68.6	77.9
2013	11.2	12.9	28.5	31.9	7.2	8.4	8.8	9.8	4.4	4.9	4.1	4.6	68.4	77.2
2014	13.3	14.7	41.2	44.6	10.2	11.5	13.3	14.0	5.5	6.1	5.8	6.3	93.6	101.6
2015	13.6	14.7	39.6	42.3	11.5	13.1	14.5	15.5	3.8	4.4	4.8	5.2	94.0	101.9
2016	11.9	12.3	32.4	33.0	12.9	13.4	9.6	9.9	4.5	4.9	4.3	4.3	81.0	83.4

CIR, crude incidence rate; ASIR, age-specific and standardized incidence rate.

**Table 3:** Incidence temporal trends (age-standardized to the Segi standard population) for the common GI cancers and all GI neoplasms combined by sex in Wuwei, northwestern China, 1995–2016.

Site		Trend 1			Trend 2			Trend 3		
		Years	APC	95%CI	Years	APC	95%CI	Years	APC	95%CI
Esophagus	Male	1995–1997	122.9	-30.7–616.9	1997–2009	8.7*	5.2–12.3	2009–2016	-4.2	-9.2–1.1
	Female	1995–2016	3.7*	0.6–6.8						
	Total	1995–2009	9.4*	5.4–13.6	2009–2016	-4.6	-11.5–2.8			
Stomach	Male	1995–2009	12.9*	9.8–16.1	2009–2016	4.0	-0.5–8.7			
	Female	1995–2016	8.1*	6.4–9.9						
	Total	1995–2014	10.5*	8.9–12.1	2014–2016	-12.0	-34.5–18.3			
Colon & rectum	Male	1995–2009	10.1*	6.9–13.5						
	Female	1995–2009	11.8*	9.1–14.6						
	Total	1995–2009	11.0*	8.4–13.7						
Pancreas	Male	1995–2009	9.3*	6.5–12.2						
	Female	1995–2016	12.2*	9.0–15.4						
	Total	1995–2016	11.2*	8.5–13.9						
Liver	Male	1995–2009	16.9*	12.6–21.4	2009–2016	4.0	-1.6–9.9			
	Female	1995–2016	12.3*	9.8–14.9						
	Total	1995–2009	16.7*	12.3–21.3	2009–2016	5.1	-0.6–11.1			
Gastric cardia	Male	1995–2009	10.3*	7.9–12.7	2009–2016	-18.5*	-13.8–7.7			
	Female	1995–2009	8.5*	4.0–13.2	2009–2016	-12.7*	-21.3–3.2			
	Total	1995–2009	9.9*	7.4–12.4	2009–2016	-17.3*	-22.0–12.3			
All sites combined	Male	1995–2009	12.4*	10.1–14.8	2009–2016	0.3	-3.3–3.9			
	Female	1995–2016	8.9*	7.2–10.6						
	Total	1995–2009	12.2*	9.8–14.6	2009–2016	1.4	-2.3–5.1			

APC, annual percentage change; *The APC is significantly different from zero (two-side P < 0.05); CI, confidence interval.

**Figure 2:**
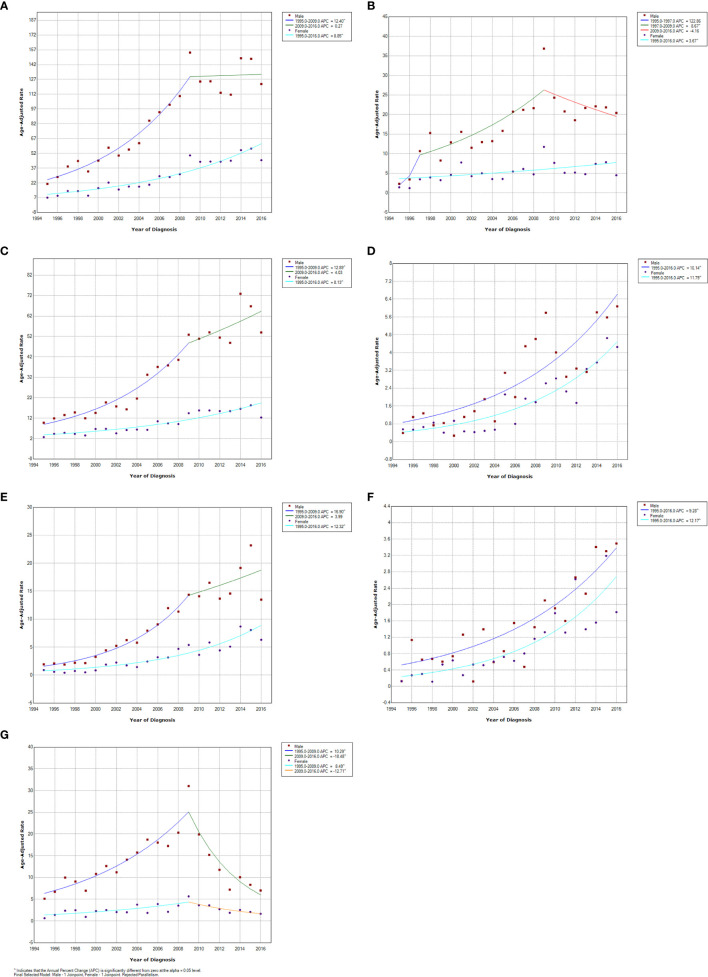
Trend of annual percentage change for the age-adjusted standardized incidence rates of all gastrointestinal (GI) cancers and the common GI cancers stratified by sex in Wuwei, northwestern China, 1995–2016. **(A)** all GI cancers, **(B)** Esophageal cancer, **(C)** Gastric cancer, **(D)** Colorectal cancer, **(E)** Liver cancer, **(F)** Pancreatic cancer, **(G)** Gastric cardia cancer.

**Figure 3:**
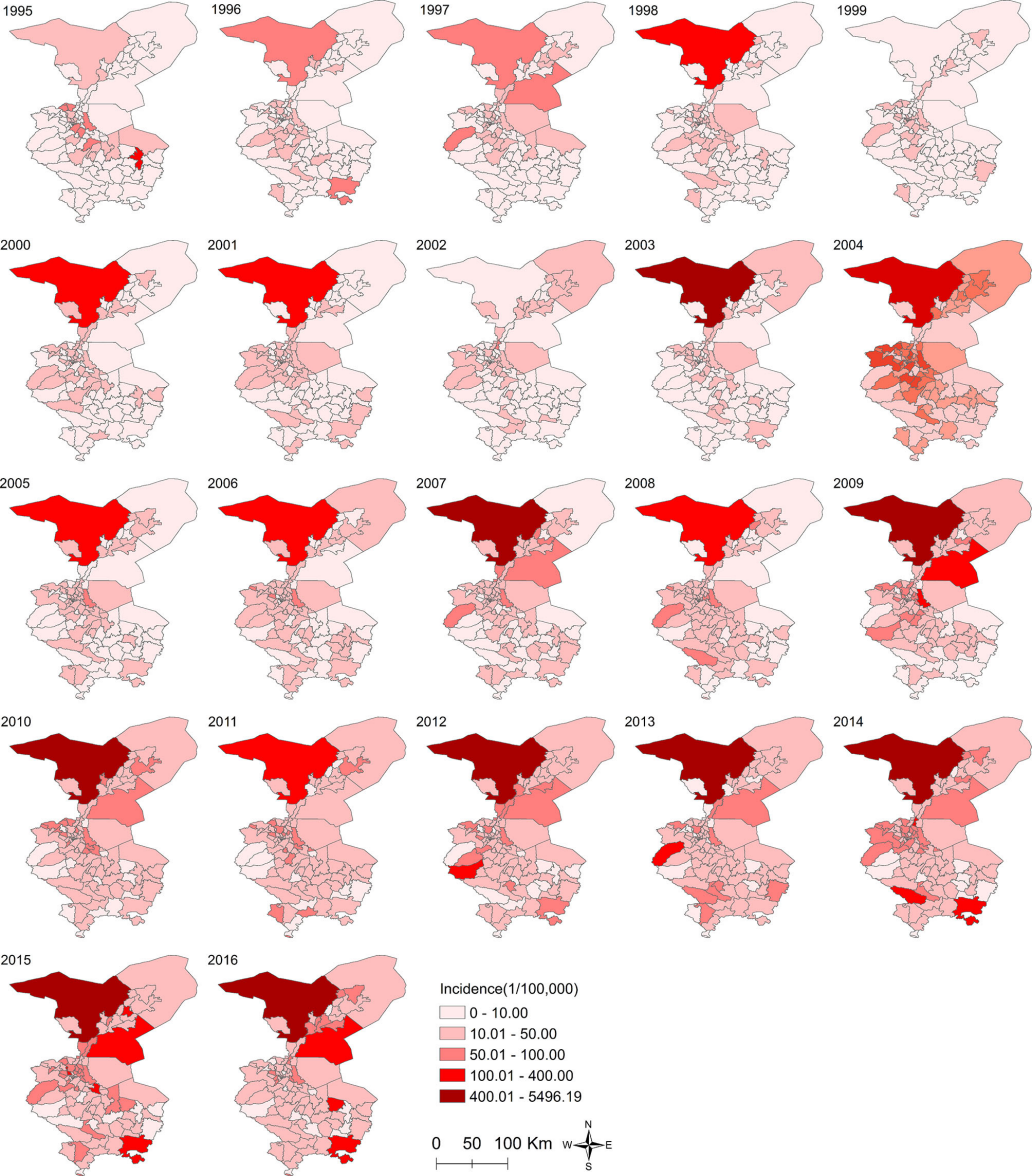
The annual crude incidence rates of all gastrointestinal (GI) cancers among the townships in Wuwei, northwestern China, 1995–2016.

The trends of overall and sex-specific APCs of the ASIRs for all GI neoplasms and six major GI cancers during the study period are shown in **Figures 2A–G**. With the geographical distribution maps, the annual CIRs of all GI neoplasms are shown in **Figure 3** and six GI cancers during the study period are shown in **Figures S2A–F**. Due to the long timespan, the study was divided into four time periods and the geographical distribution maps of the average incidence of each time period were created to better reveal the variation of temporal and spatial distribution of the GI neoplasms in Wuwei (**Figures S3A–F**).

### Esophageal Cancer

Esophageal cancer, with an overall ASIR of 11.00 per 100,000 person-years, had the highest increase in ASIR from 1.86 to 24.33 per 100,000 person-years between 1995 and 2009, and then showed a steady decrease at the rate of 4.16 per 100,000 person-years per year from 2009 to 2016 (**Table 2**, **Figure 2B**). Both sexes showed a similar pattern, and male patients had a higher ASIR within the study timeframe. The numbers of esophageal cancer patients increased by 9.4% per year during 1995 to 2009 and 8.7% per year from 1997 to 2009 in males, and the rate increased by 3.7% per year during the entire study period in females. There were significant differences in the annual CIRs among the townships in Wuwei; however, no significant temporal change in the CIR for each township was observed during the study period (**Figure S2A**).

### Gastric Cancer

Gastric cancer represented the leading cause of morbidity with an ASIR of 21.81 per 100,000 person-years from 1995 to 2016 (**Table 2**, **Figure 2C**). After a steady increase from 1995 to 2014, the overall ASIR of gastric cancer reached a peak value recorded in 2014 with an ASIR of 44.61 per 100,000 person-years (**Figure 2C**). A similar pattern was seen for the sex-specific ASIR of gastric cancer although males had a significantly higher incidence than females throughout the study period (**Figure 2C**). However, male patients increased about 2.5 times more than that of female cases from 2010 to 2014 (**Tables 2**, **3**). The geographic distribution map showed that patients with gastric cancer increased in almost all townships, with a higher incidence existed in the northern areas (**Figure S2B**).

### Colorectal Cancer

Colorectal cancer had an ASIR of 5.71 per 100,000 person-years, and an increasing linear trend in the overall ASIR was observed during the study period (**Tables 2**, **3**). There was a significant increase in the ASIR of colorectal cancers in males and females from 1995 to 2009, with 10.1 and 11.8 per 100,000 person-years, respectively (**Table 3**, **Figure 2D**). The incidence increased from 1.1 to 13.7 per 100,000 person-years in males, projecting to arrive at 0.8 per 100,000 person-years, and increased from 0.8 to 13.1 per 100,000 person-years in females during the study period. The geographic distribution map showed that patients with colorectal cancer increased in almost all townships, with a higher incidence in the northern areas (**Figure S2C**).

### Liver Cancer

The overall ASIR of liver cancer was 6.28 per 100,000 person-years from 1995 to 2016 (**Table 2**). The ASIR showed a sustained annual growth from 1995 to 2008 and then a sharp increase between 2008 and 2009 that that mostly plateaued after 2009 (**Table 3**, **Figure 2E**). Sex-specific time trend for liver cancer incidence was almost the same as the overall trend of the disease. As expected, males had a much higher morbidity than females during the study period, particularly since 1995, and maintained an upward trend until a peak in 2015 with an ASIR of 15.5 per 100,000 person-years (**Tables 2**, **3**, **Figure 2E**). The thematic maps of the annual incidence rate at the township level demonstrated that the area of high incidence was gradually expanding, especially in Liangzhou District of central Wuwei (**Figure S2D**).

### Pancreatic Cancer

The overall ASIR of pancreatic cancer was 1.83 per 100,000 person-years between 1995 and 2016 (**Table 2**). The ASIR showed an upward trend from 0.1 per 100,000 person-years in 1995 to 6.1 per 100,000 person-years in 2014 which was a 60-fold absolute increase although it showed a slight decline in 1999 and 2000 (**Table 3**, **Figure 2F**). The ASIR increased by 11.2% per year during the study period and peaked in 2014 with an ASIR of 6.1 per 100,000 person-years. The geographic distribution of the annual incidence showed that only one township in the northernmost area showed a high incidence from 2013 to 2016 (**Figure S2E**).

### Gastric Cardia Cancer

The overall ASIR of gastric cardia cancer was 7.82 per 100,000 person-years, between 1995 and 2016 (**Table 2**). In 1995, the ASIR for gastric cardia cancer was 2.9 per 100,000 person-years, and increased to a peak value of 18.4 per 100,000 person-years in 2009 with an APC of 9.9 per year. Then, the ASIR significantly decreased by 17.3% per year, with a greater decrease in males than in females (18.5% *vs* 12.7%) (**Table 3**, **Figure 2G**). The thematic map showed that the geographic distribution of gastric cardia cancer was similar to that of gastric cancer, and the former existed a higher population incidence in the northern city (**Figure S2F**).

## Discussion

In this study, the ASIRs of six major GI neoplasms generally showed a steep upward trend, and the ASIRs were significantly higher in males than in females in Wuwei, northwestern China, from 1995 to 2016. The ASIRs for all GI neoplasms significantly increased by 12.2% per year from 1995 to 2009 and by 1.4% per year from 2009 to 2016 while the latter increase was not significant. However, the ASIRs significantly increased for all individual GI neoplasms from 1995 to 2009, but varied in the trends from increasing by 8.7% per year for esophageal cancer to decreasing by 12.7% per year for gastric cardia cancer from 2009 to 2016. Moreover, the annual incidence of all GI neoplasms showed varying degrees of differential distribution among the townships of Wuwei. Before 2009, the ASIRs for all GI neoplasms and all individual GI neoplasms in Wuwei has been on the rise. In 2009, the National Health and Family Planning Commission conducted an early diagnosis and early treatment screening for upper GI cancers in Wuwei City with special funds from the central government, which helped change the ASIRs for GI neoplasms thereafter (14).

An uptrend was detected in the present study during the periods from 1995 to 2014, which was not in accordance with previous studies in China and other countries (15–17). While gastric cancer is the fourth most common type of cancer and the second leading cause of cancer-related deaths worldwide (18, 19), the overall incidence for gastric cancer has steadily fallen in many countries during the past few decades (11–13). The decline in the incidence in most countries and regions can be explained by the easy access to health care, preventive services, and reduction in exposure to the known risk factors, such as *Helicobacter pylori* (*H. pylori*) infection, smoking, drinking alcohol, excessive intake of salt, and consumption of salt-preserved or nitrous-containing foods (5, 13, 18, 20–23). Balakrishnan et al. reported that the initial decrease in gastric cancer incidence was associated with changes in food preservation and availability (low salt, low nitrite), especially of fresh fruits and vegetables, followed by a decrease in the primary etiologic factor, H. pylori (24). Similarly, Kim SR et al. found that an increase of white meat consumption may reduce the risk of gastric cancer, while red or processed meat may increase the risk of gastric cancer (25). It has been reported that the population living in Wuwei prefer to eat red and processed meat that has been salted or fried and are less likely to eat fresh vegetables, fruits, fish, and milk (26), which might result in the increasing trend of ASIRs of gastric cancer. Moreover, previous studies demonstrated the presence of many strong carcinogens in the diet of Wuwei residents, such as cancer-promoting fungi and volatile N-nitroso compounds (27) which might also accelerate the increasing trend. Our results showed that the ASIR of male patients was higher than females which is consistent with the studies in other countries (28). However, there is no consensus on effective strategies for identifying target groups and an appropriate age for screening potential sufferers (29). It is suggested that risk stratification approaches be used for high-risk individuals in order to offer appropriate screening strategies and to control the burden of disease in certain areas (30).

Consistent with the global growth pattern, a steep increase in morbidity for esophageal cancer was found in Wuwei since 1995. This trend might be attributed to changes in the environment and lifestyle of Chinese people. Possible factors involved in these changes include drinking unhygienic water, unhealthy food preparation methods, smoking, drinking hot tea, and opium use in Northern China, as suggested in previous studies (31, 32). Although new advances in the diagnosis technique and clinical medicine have been developed in recent decades, which helped screen for early esophageal cancer and treat it accordingly, due to the high cost of endoscopic screening, high requirements for personnel, and the lack of awareness of local people about their own health, the current coverage of screening population is limited (33).

Usually, the high prevalence of gastric cardia cancer is paralleled by the high incidence of esophageal and gastric cancer (34). The present study revealed that the geographical distribution and high-risk population of gastric cardia cancer in Wuwei were similar for that of esophageal and gastric cancer; however, the temporal trends of gastric cardia cancer were significantly different from whose of the esophageal and gastric cancer, which fell fast from 2009. We speculated that this might be related to their etiological differences. A previous epidemiological study demonstrated a strong association between an increased body mass index and the risk of gastric cardia cancer development (35), which are not associated with gastric and esophageal cancer. Long-term studies with large samples are needed to identify the reasons behind the disparities in the future.

In the present study, contrary to the condition in the United States and some well-developed Asian countries, the incidence of colorectal cancer showed a steeply upward trend in Wuwei during the study period. It has been widely accepted that colorectal cancer is a disease correlated with economic status. There are evidences supporting a close association between colorectal cancer risk and certain lifestyle behaviors and diseases, including sedentary lifestyle, unhealthy diet, high-calorie/fat diets, alcohol consumption, smoking, physical inactivity, and obesity, which are worsening in China (4, 36). The increasing incidence of colorectal cancer could also be attributed to an easy access to advanced screening, diagnoses, and other medical facilities in urban areas. The rising incidence of colorectal cancer in Wuwei City might also be due to the increased attention to health by patients. People would like to go to the doctor in advance when they find that they are not well, which increases the detection rate of the disease. With the development of the medical insurance policies, the problem of a difficult and expensive medical treatment has been solved to some extent, which also increases the diagnosis rate of colorectal cancer. However, previous studies demonstrated the decreasing trend in colorectal cancer mortality is likely due to the enhanced access to screening, colonoscopy-early stage detection, and new advances in treatments (37, 38), which was contrary to the results of our study and other studies in China. Li found that the standard incidence of colorectal cancer in both men and women in Shanghai showed an upward trend, and the upward trend of colon cancer was more significant than that of rectal cancer (39). Further studies are needed to explore the possible factors associated with the increasing morbidity for colorectal cancer in China in recent decades.

Our study revealed a significant increase in the incidence of pancreatic cancer during the last two decades, consistent with a previous study that the age-standardized incidence and mortality of pancreatic cancer have increased by 49.88% and 47.51% during 1990 and 2017, respectively (40). The increasing trend could be due to the development of living standard and urbanization. Pancreatic cancer is thought to be associated with the human development index (41). The high incidence and mortality of pancreatic cancer are commonly reported in developed countries. Statistical institutions in different regions worldwide have witnessed increasing trends in the mortality for pancreatic cancer, possibly due to the advances in diagnostic technology, such as biopsy guided by computed tomography, endoscopic ultrasonography, or biomolecular reagent while those sufferers are usually diagnosed at advanced stages because of natural history and lack of screening (42). Our results illustrated that the incidence was significantly higher in males than in females, which is consistent with the growth patterns reported from Southern Europe and North East Asia (43). Moreover, tobacco smoking, as an important risk factor for pancreatic cancer, likely explains the international and sex-specific differences (43). In China, about two-thirds of the men smoke, and it is likely that smoking along with other risk factors, such as advancing age, might account for the increasing mortality for pancreatic cancer in the Chinese male group (43, 44). A previous study on the lifestyle of the Wuwei population showed that the proportion of male smokers was about 50 times higher than that of the females (45). Moreover, some studies have also reported that environment pollution raises the risk of mortality, especially in older population, which could partially explain the rising tendency of pancreatic cancer in the recent years (46, 47).

Liver cancer, one of the leading causes of cancer death worldwide, is the second-most common cancer in China (48). Our results showed that it had a lower ASIR compared with other GI neoplasms in Wuwei; however, the morbidity of liver cancer increased significantly from 1995 to 2016. Moreover, the morbidity of liver cancer in the male and female populations was increased significantly compared with the results of previously published studies (49). In China, most cases of liver cancer are attributable to hepatitis B virus infection (4, 5). More reduction in liver cancer mortality is expected in the coming decades as an outcome of widespread hepatitis B vaccination, which has reached >98% coverage in infants in 2015 (48). However, HBV infection is still common at present, possibly because the majority of Chinese adult sufferers were born before the implementation of the national hepatitis B vaccination for neonates in 1994. Moreover, nonalcoholic fatty liver disease is another one of the leading causes of cirrhosis that leads to liver cancer, which might partly explain the increasing incidence for liver cancer in China and calls for public health strategies and concrete actions.

Several limitations should be acknowledged. Firstly, the data were obtained from a passive surveillance system from 12 major public hospitals, which may be the major limitation. Some shortcomings may arise from possible inconsistencies in cancer diagnosis and unavoidable under-reporting or incompleteness in our coverage of the population (50). Secondly, the proportion of known diagnosis *versus* unidentified causes of death has increased over time for patients with liver cancer. These might, in part, contribute to the observed increases in incidence in some GI neoplasms. Therefore, the current results, particularly for liver cancer incidence, should be interpreted with caution. Thirdly, some liver tumors in the clinical practice are metastatic, which mainly originates in the digestive tract, but may be diagnosed as primary hepatocellular cancer when liver biopsy is not accessible or not possible, and thus the metastatic nature was not specified in the death certification. Therefore, further large-scale studies supported by examination methods of gold standard are needed in the future.

## Conclusions

In conclusion, there were comprehensive and updated trends of in-hospital incidence for GI neoplasms in Wuwei, northwestern China, from 1995 to 2016, and significant increases in the incidence of hospitalization for GI neoplasms including gastric, esophageal, colorectal, liver, gallbladder, and gastric cardia cancer. These results provide new insights into the incidence of GI diseases to policymakers and clinicians, and highlight evolving disease trends and the need for health policy planning and resource reallocation. Further studies are needed to figure out whether other cities also show the same increasing trend of GI neoplasms, and the potential pathogenic mechanism and influence factors, so that China CDC and the local CDC of other cities could adopt effective measures to contain this trend.

## Data Availability Statement

The raw data supporting the conclusions of this article will be made available by the authors, without undue reservation.

## Ethics Statement

The studies involving human participants were reviewed and approved by the Xi ‘an Center for Disease Control and Prevention Ethics Review Committee. Written informed consent for participation was not required for this study in accordance with the national legislation and the institutional requirements.

## Author Contributions

KL and ZS conceived the idea. TF, HZ, WZ, SS, ZH, and ZL collected the data. KL, YL, MY, and HS analyzed the data. KL, ZJ, and SS wrote the manuscript. All authors contributed to the article and approved the submitted version.

## Funding

This work was supported by the National Natural Science Foundation of China (81803289, 81773488), China Special Grant for the Prevention and Control of Infection Diseases (2017ZX10105011), and the Natural Science Foundation of Shaanxi Province (2020JM-329). The funding agencies had no role in the study design, data collection and analysis, or preparation of the manuscript.

## Conflict of Interest

The authors declare that the research was conducted in the absence of any commercial or financial relationships that could be construed as a potential conflict of interest.

## Publisher’s Note

All claims expressed in this article are solely those of the authors and do not necessarily represent those of their affiliated organizations, or those of the publisher, the editors and the reviewers. Any product that may be evaluated in this article, or claim that may be made by its manufacturer, is not guaranteed or endorsed by the publisher.

## Abbreviations

GI, gastrointestinal; ICD-10, International classification of Diseases Tenth revision; APC, annual percentage change; ASIRs, age-standardized incidence rates; CIRs, crude incidence rates.
