# Supplementary material for: Spatiotemporal Trends in the Incidence of Gastrointestinal Neoplasms in Wuwei City of Northwestern China From 1995 to 2016: A Hospital-Based Retrospective Observational Study
Source: Front Oncol. 2021 Sep 3;11:712857. doi: 10.3389/fonc.2021.712857 (PMC8452457; doi:10.3389/fonc.2021.712857)
Supplement: Supplementary file 1 [file DataSheet_1.pdf]

Supplemental Materials

Spatiotemporal Trends in the incidence of gastrointestinal neoplasms in Wuwei city of northwestern China from 1995 to 2016: A hospital-based retrospective observational study

Kun Liu, Shuxuan Song, Yiwen Liu, Hui Zhang, Ting Fu, Min Yan, Zhen He, Weilu Zhang, Haixia Su, Zhao Li, Zhaohua Ji, Zhongjun Shao

Figure of Contents

Figure S1 **The location of study area and the hospitals.**

Figure S2 **The annual crude incidence rates of the common GI cancers among the townships in Wuwei, Northwestern China, 1995-2016.** A: Esophageal cancer, B: Gastric cancer, C: Colorectal cancer, D: Liver cancer, E: Pancreatic cancer, F: Gastric cardia cancer.

Figure S3 **The spatiotemporal distribution of the six common GI cancers in four time period in Wuwei, Northwestern China, 1995-2016.** A: Esophageal cancer, B: Gastric cancer, C: Colorectal cancer, D: Liver cancer, E: Pancreatic cancer, F: Gastric cardia cancer.

Figure S1

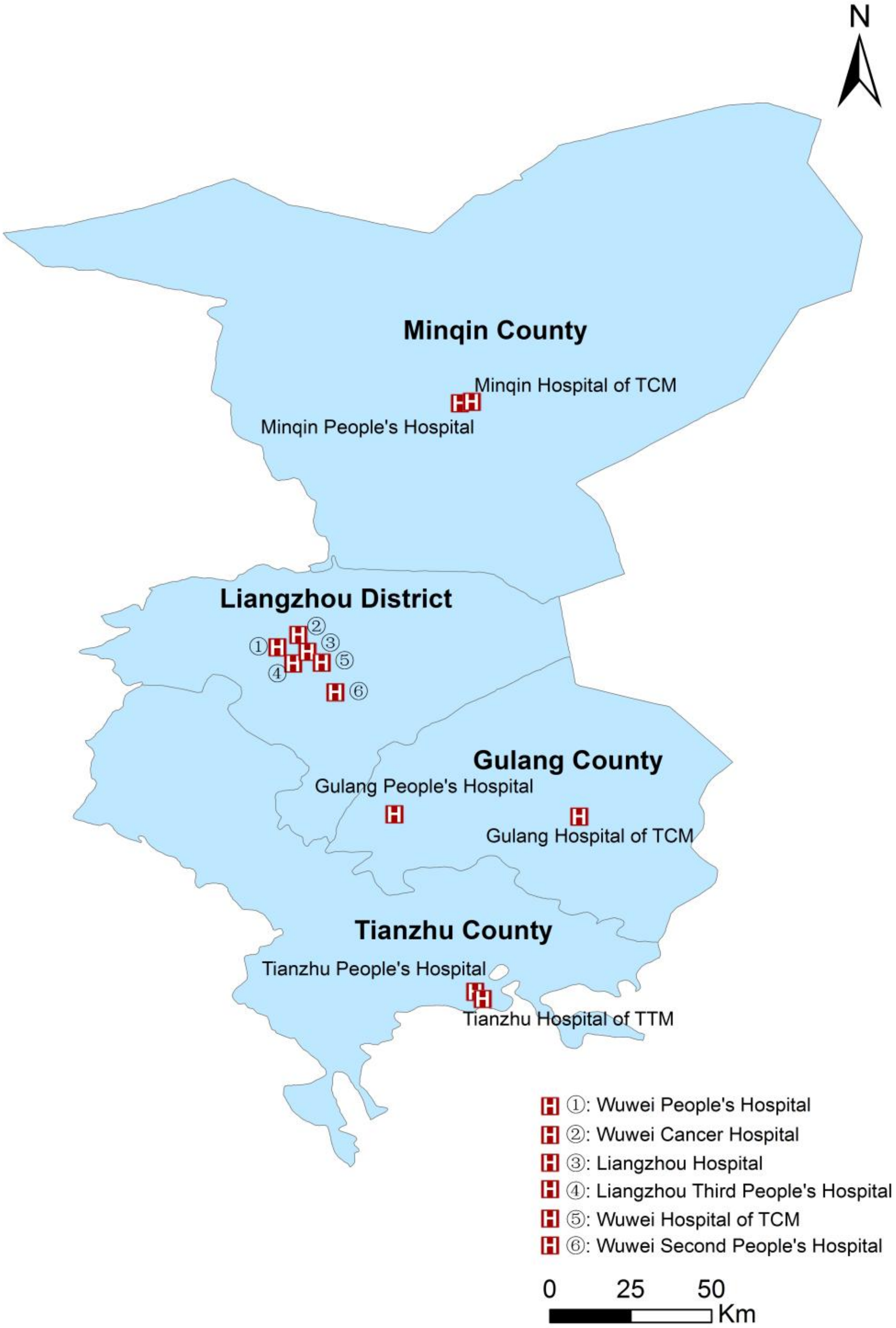
N
Minqin County
Minqin Hospital of TCM
Minqin People's Hospital
Liangzhou District
①
②
③
④
⑤
⑥
Gulang County
Gulang People's Hospital
Gulang Hospital of TCM
Tianzhu County
Tianzhu People's Hospital
Tianzhu Hospital of TTM
①: Wuwei People's Hospital
②: Wuwei Cancer Hospital
③: Liangzhou Hospital
④: Liangzhou Third People's Hospital
⑤: Wuwei Hospital of TCM
⑥: Wuwei Second People's Hospital
0 25 50
Km

Figure S2-A

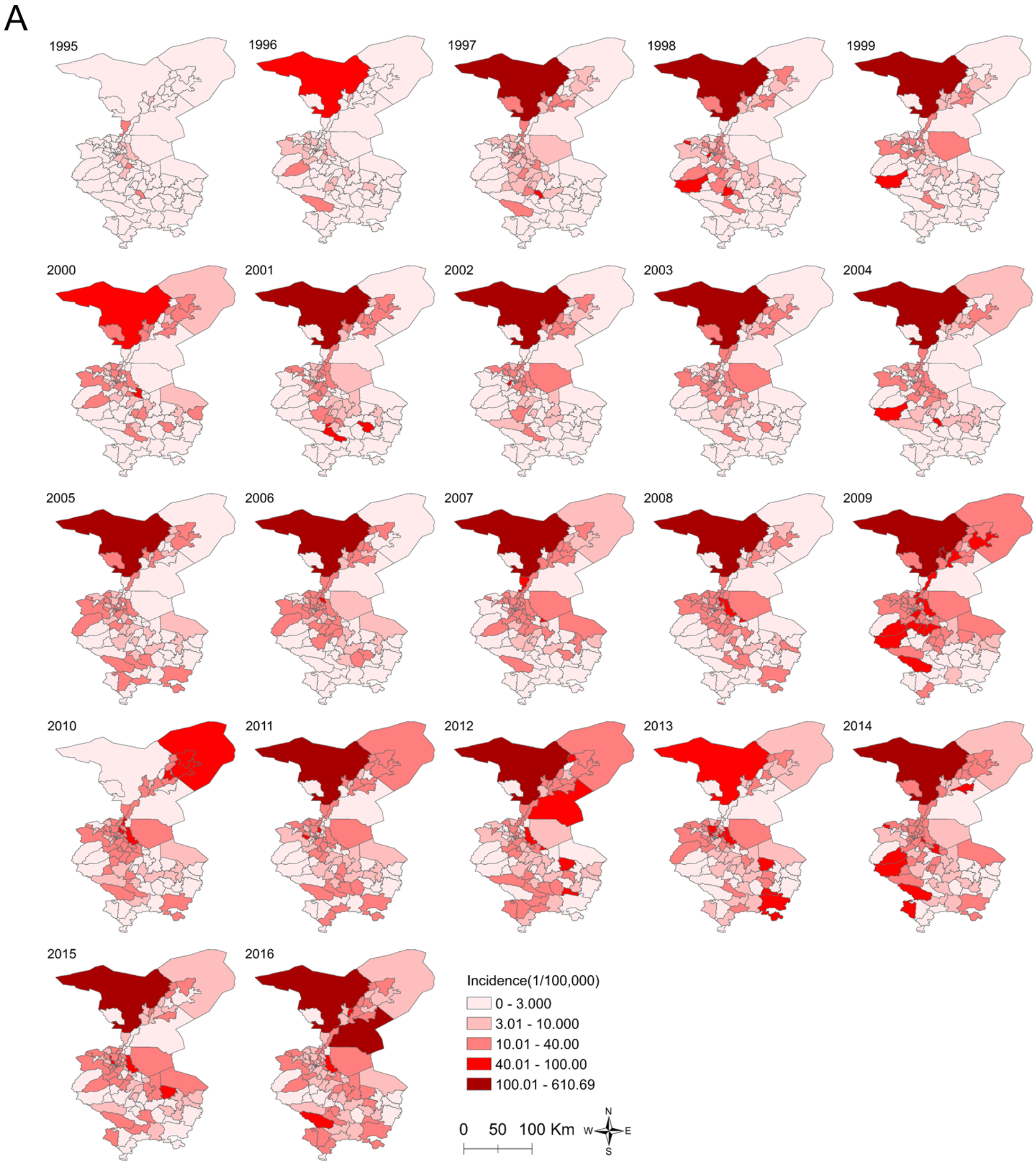
A
1995
1996
1997
1998
1999
2000
2001
2002
2003
2004
2005
2006
2007
2008
2009
2010
2011
2012
2013
2014
2015
2016
Incidence(1/100,000)
0 - 3.000
3.01 - 10.000
10.01 - 40.00
40.01 - 100.00
100.01 - 610.69
0 50 100 Km
N
W
E
S

Figure S2-B

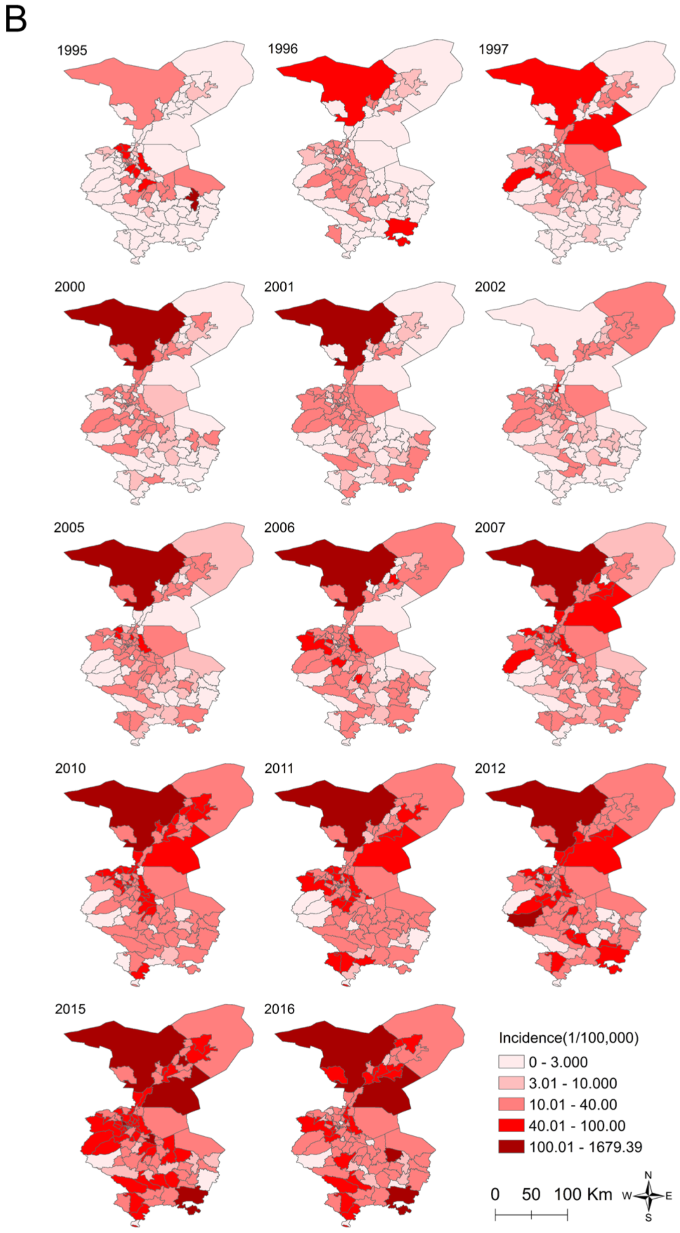
B
1995
1996
1997
1998
1999
2000
2001
2002
2003
2004
2005
2006
2007
2008
2009
2010
2011
2012
2013
2014
2015
2016
Incidence(1/100,000)
0 - 3.000
3.01 - 10.000
10.01 - 40.00
40.01 - 100.00
100.01 - 1679.39
0 50 100 Km
N
W
E
S

Figure S2-C

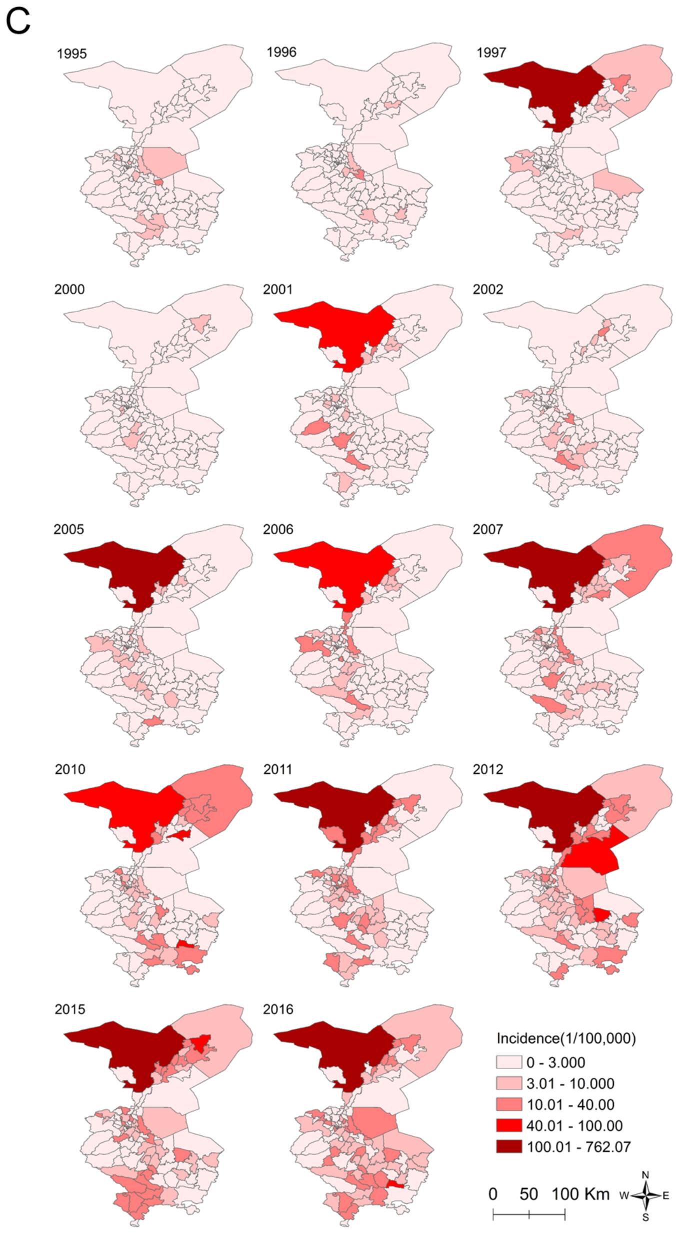
C
1995
1996
1997
1998
1999
2000
2001
2002
2003
2004
2005
2006
2007
2008
2009
2010
2011
2012
2013
2014
2015
2016
Incidence(1/100,000)
0 - 3.000
3.01 - 10.000
10.01 - 40.00
40.01 - 100.00
100.01 - 762.07
0 50 100 Km
N
W
E
S

Figure S2-D

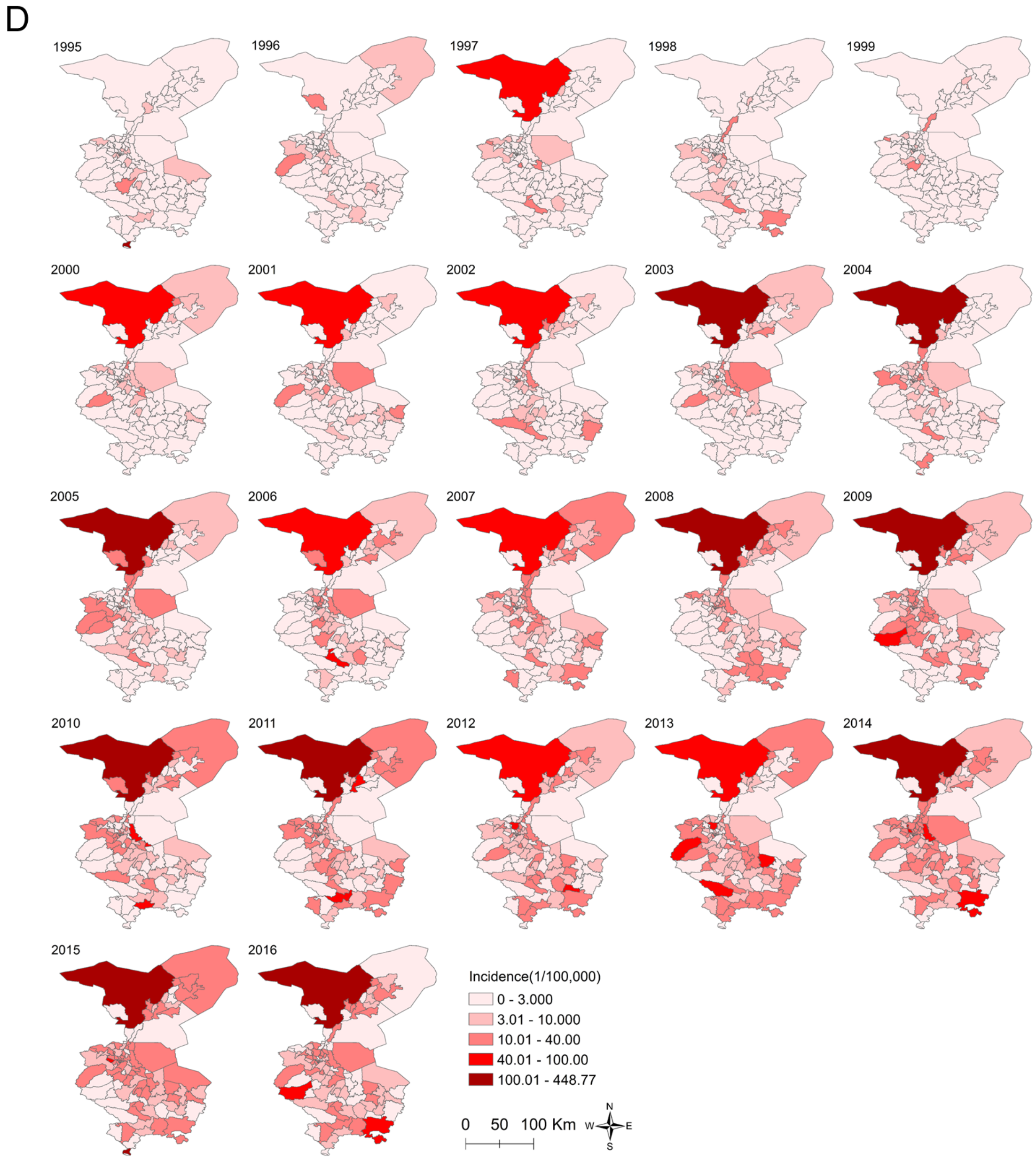
D
1995
1996
1997
1998
1999
2000
2001
2002
2003
2004
2005
2006
2007
2008
2009
2010
2011
2012
2013
2014
2015
2016
Incidence(1/100,000)
0 - 3.000
3.01 - 10.000
10.01 - 40.00
40.01 - 100.00
100.01 - 448.77
0 50 100 Km
N
W
E
S

Figure S2-E

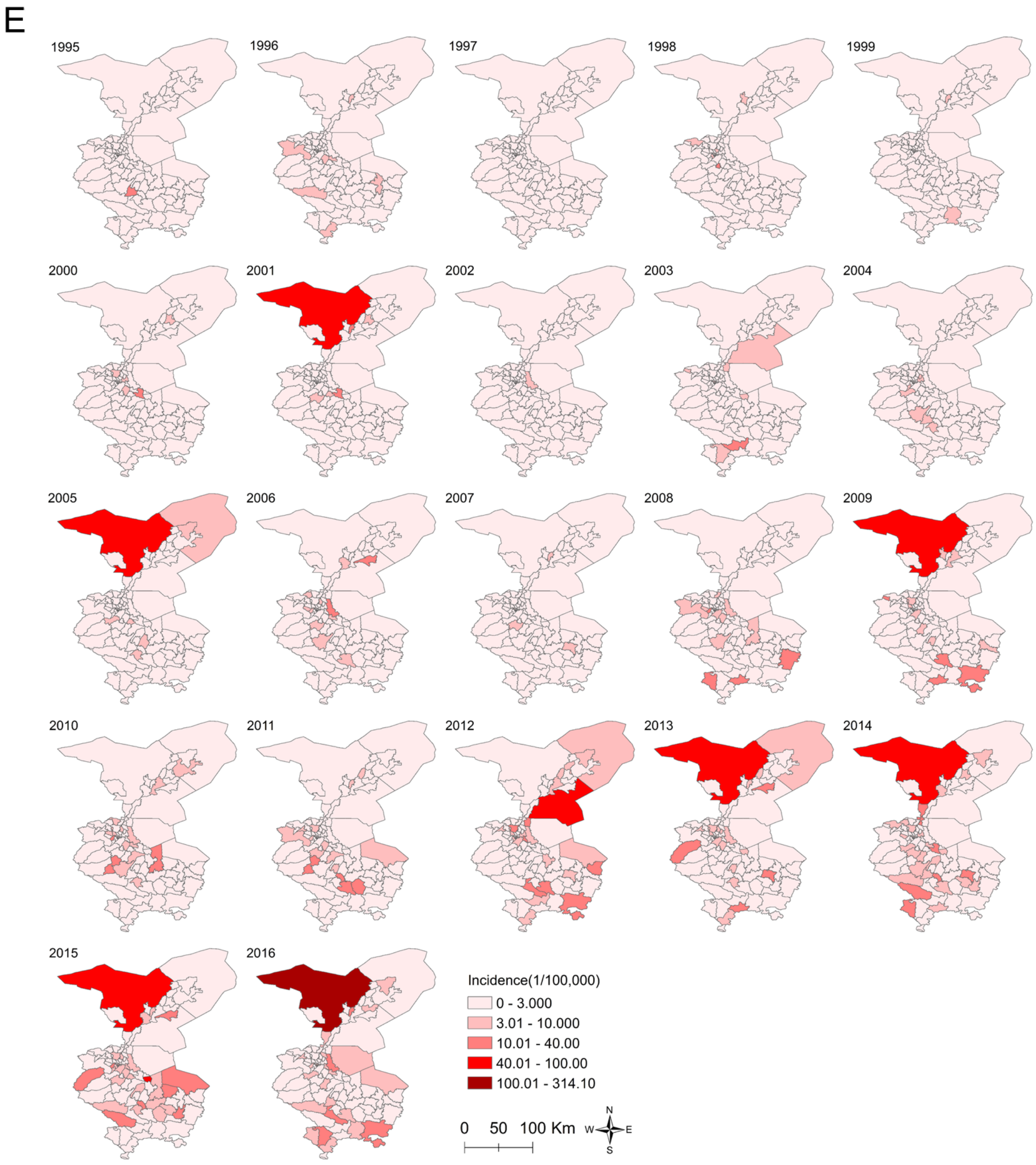
E
1995
1996
1997
1998
1999
2000
2001
2002
2003
2004
2005
2006
2007
2008
2009
2010
2011
2012
2013
2014
2015
2016
Incidence(1/100,000)
0 - 3.000
3.01 - 10.000
10.01 - 40.00
40.01 - 100.00
100.01 - 314.10
0 50 100 Km
N
W
E
S

Figure S2-F

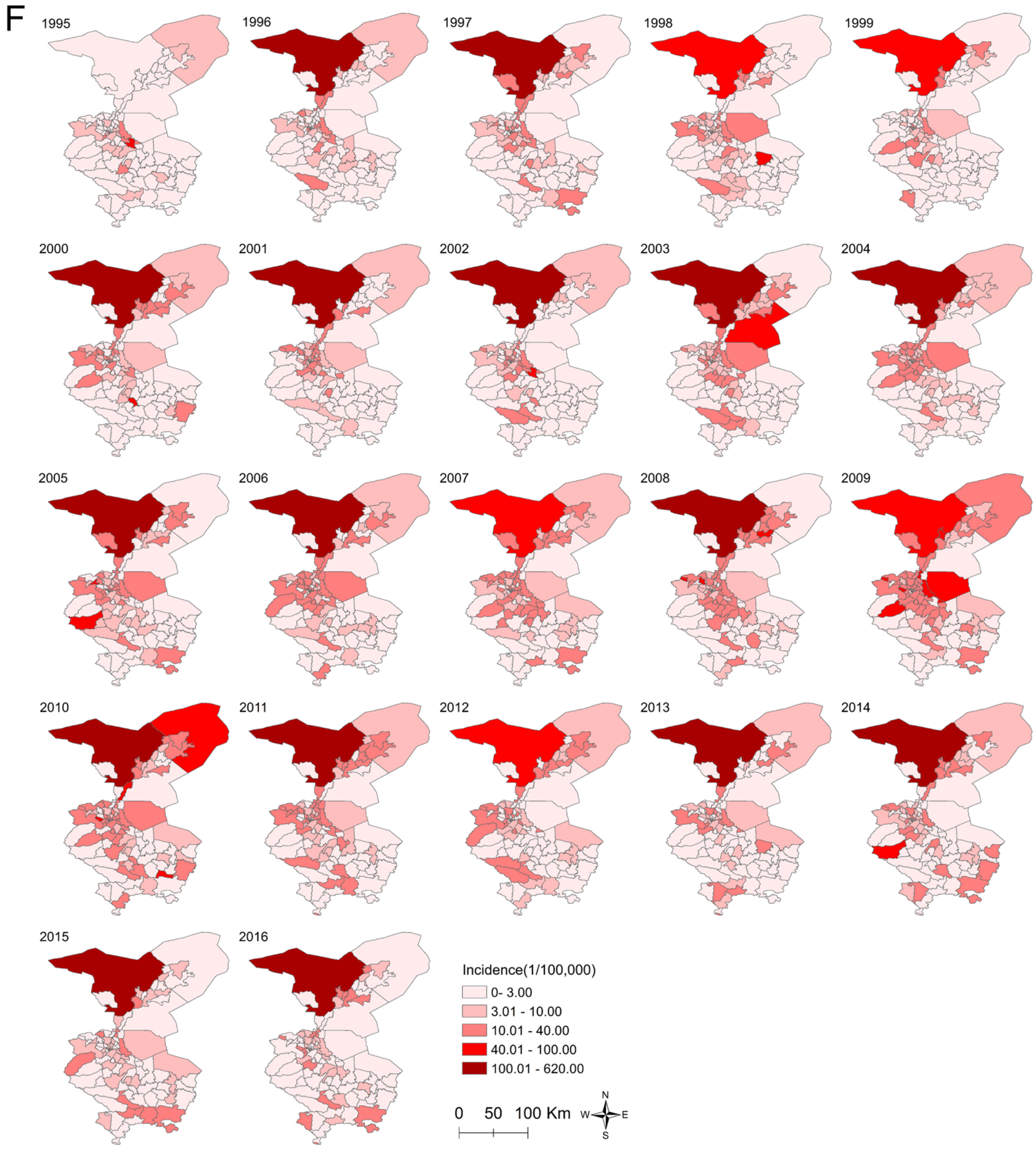
F
1995
1996
1997
1998
1999
2000
2001
2002
2003
2004
2005
2006
2007
2008
2009
2010
2011
2012
2013
2014
2015
2016
Incidence(1/100,000)
0- 3.00
3.01 - 10.00
10.01 - 40.00
40.01 - 100.00
100.01 - 620.00
0 50 100 Km
N
W
E
S

Figure S3-A

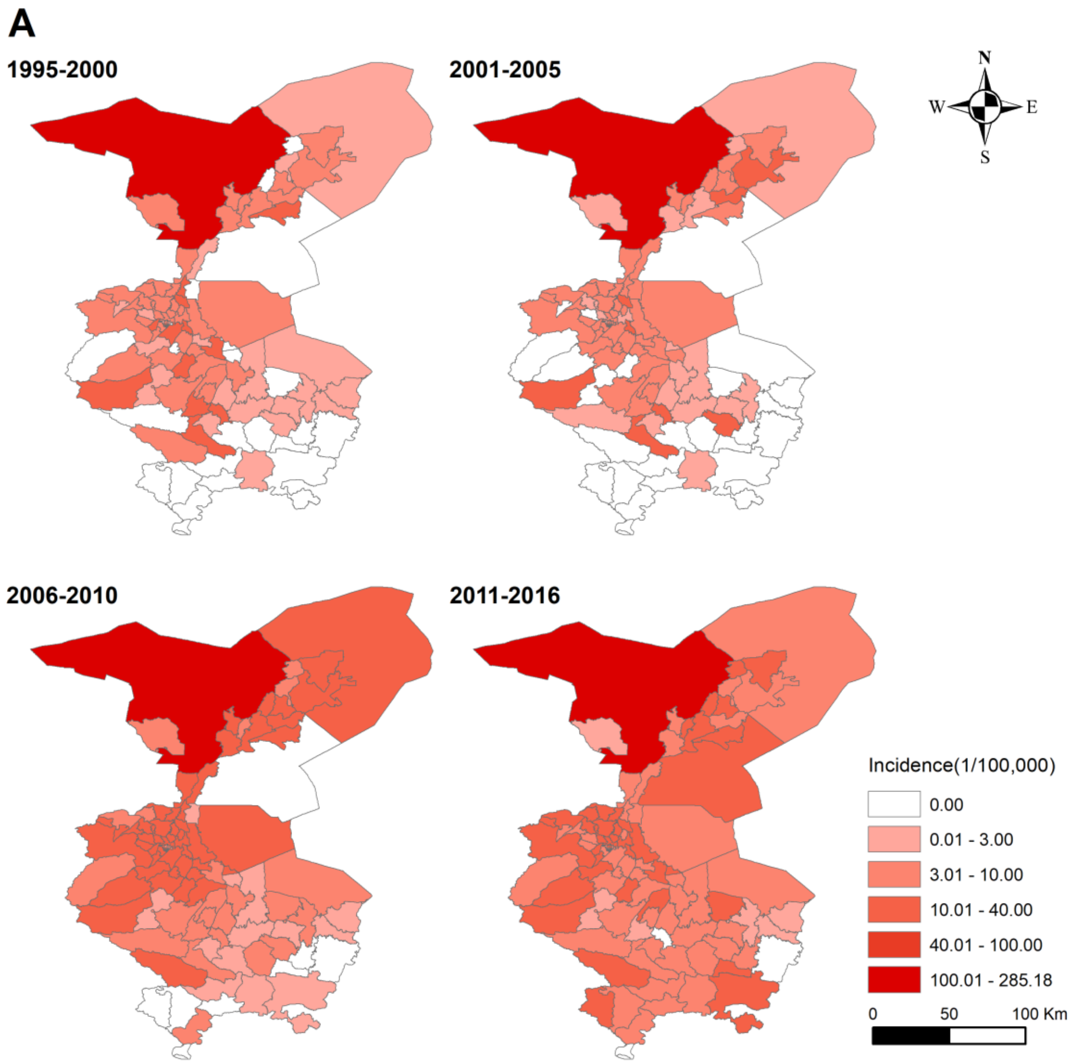
A
1995-2000
2001-2005
2006-2010
2011-2016
N
W
E
S
Incidence(1/100,000)
0.00
0.01 - 3.00
3.01 - 10.00
10.01 - 40.00
40.01 - 100.00
100.01 - 285.18
0
50
100 Km

Figure S3-B

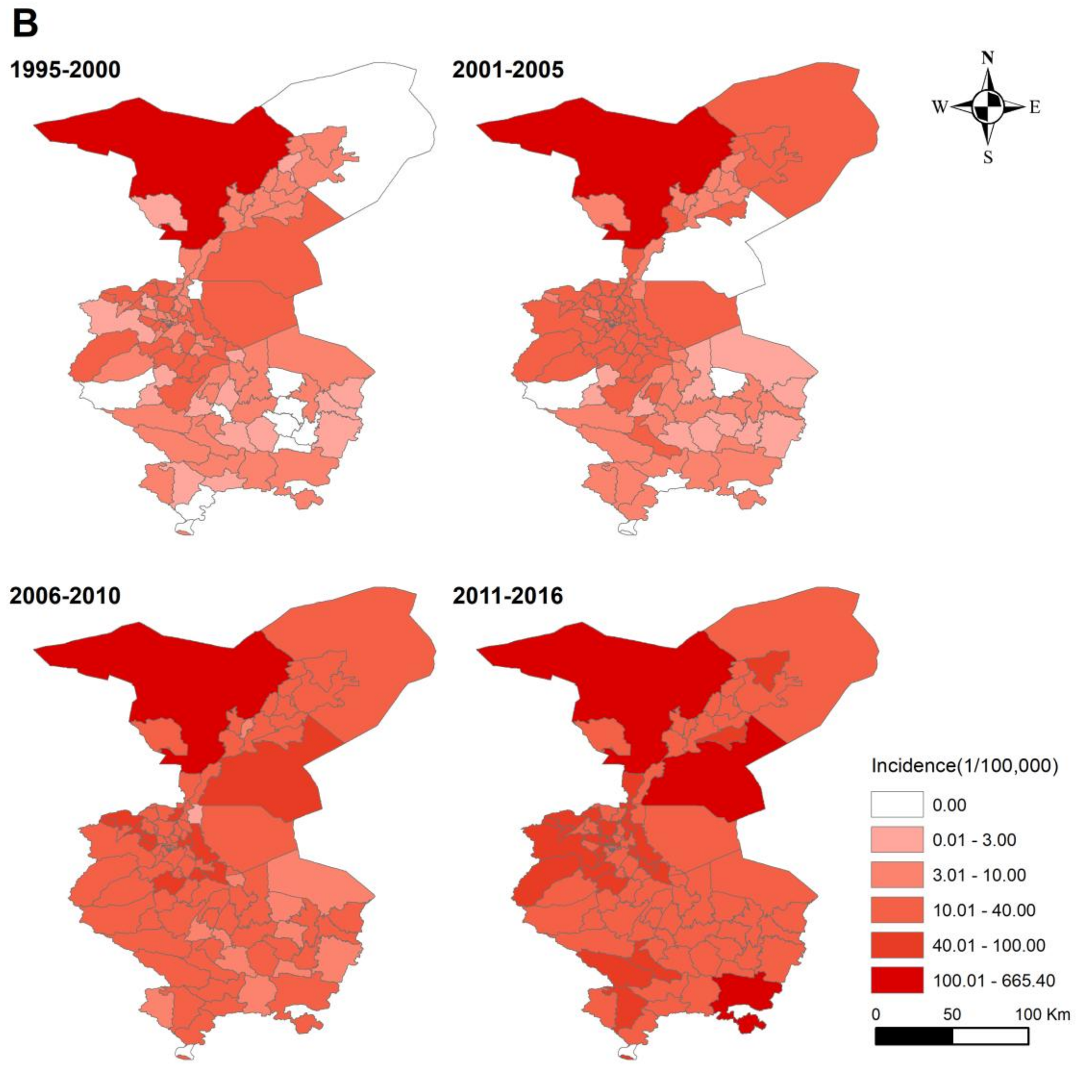
B
1995-2000
2001-2005
2006-2010
2011-2016
N
W
E
S
Incidence(1/100,000)
0.00
0.01 - 3.00
3.01 - 10.00
10.01 - 40.00
40.01 - 100.00
100.01 - 665.40
0
50
100 Km

Figure S3-C

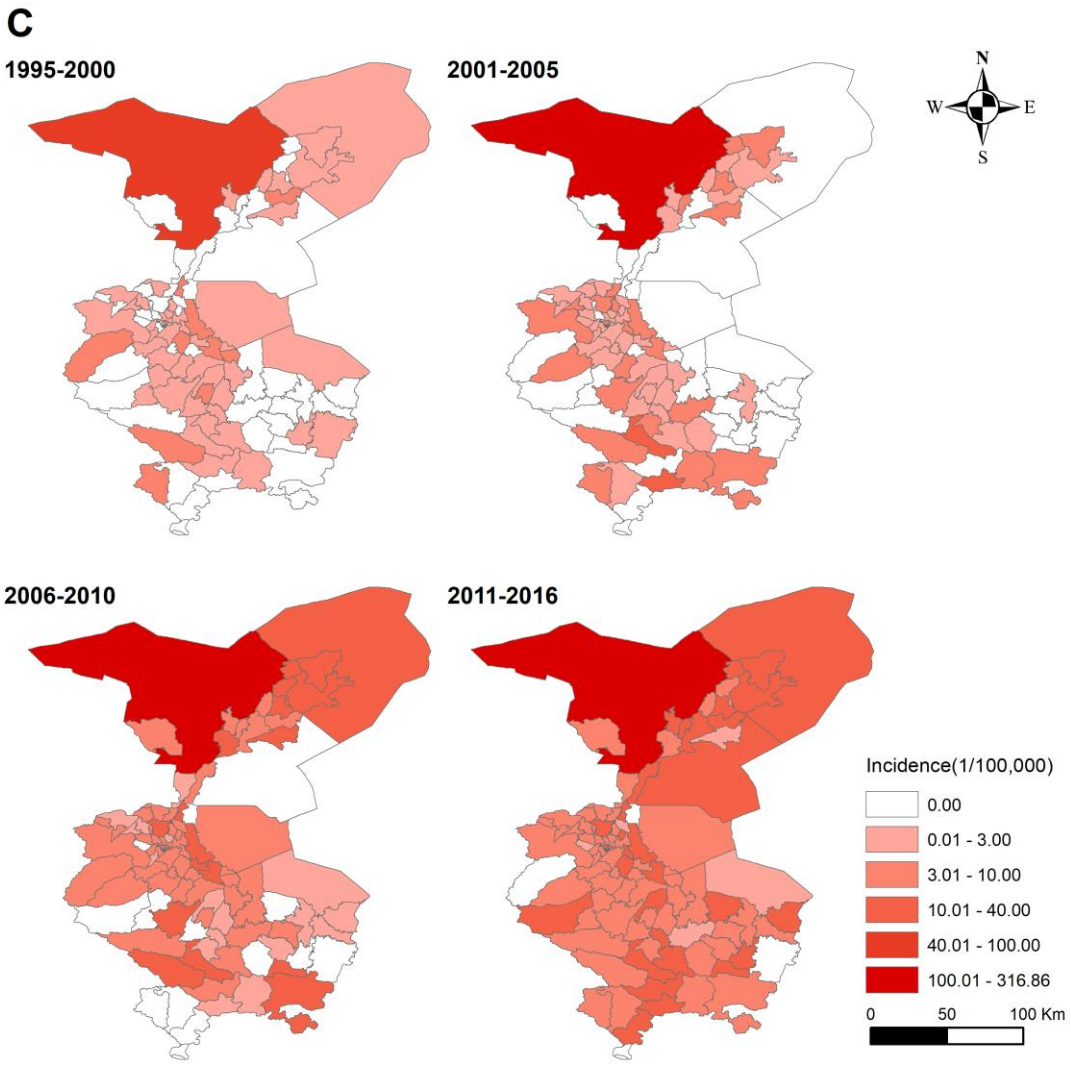
C
1995-2000
2001-2005
2006-2010
2011-2016
N
W
E
S
Incidence(1/100,000)
0.00
0.01 - 3.00
3.01 - 10.00
10.01 - 40.00
40.01 - 100.00
100.01 - 316.86
0
50
100 Km

Figure S3-D

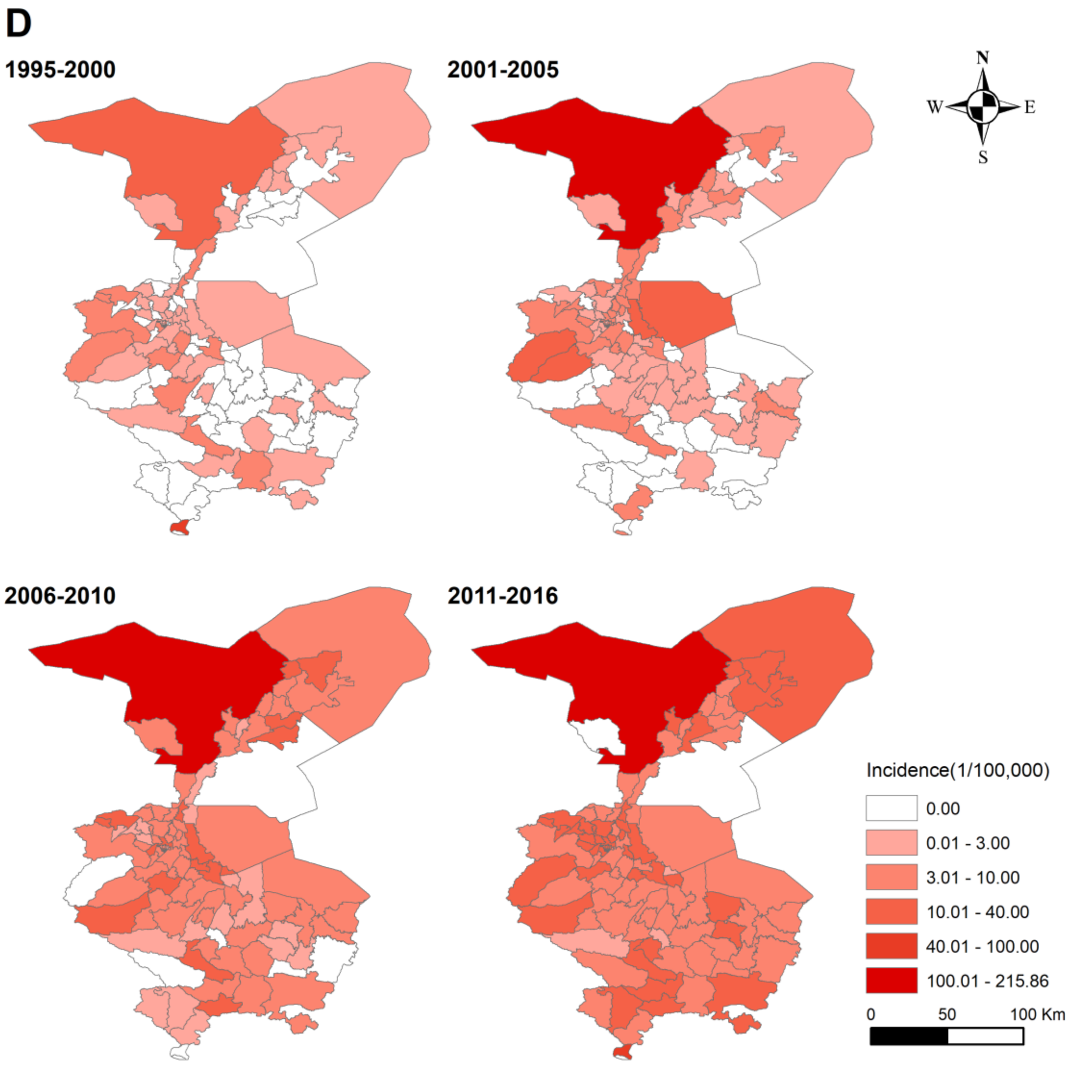
D
1995-2000
2001-2005
2006-2010
2011-2016
N
W
E
S
Incidence(1/100,000)
0.00
0.01 - 3.00
3.01 - 10.00
10.01 - 40.00
40.01 - 100.00
100.01 - 215.86
0
50
100 Km

Figure S3-E

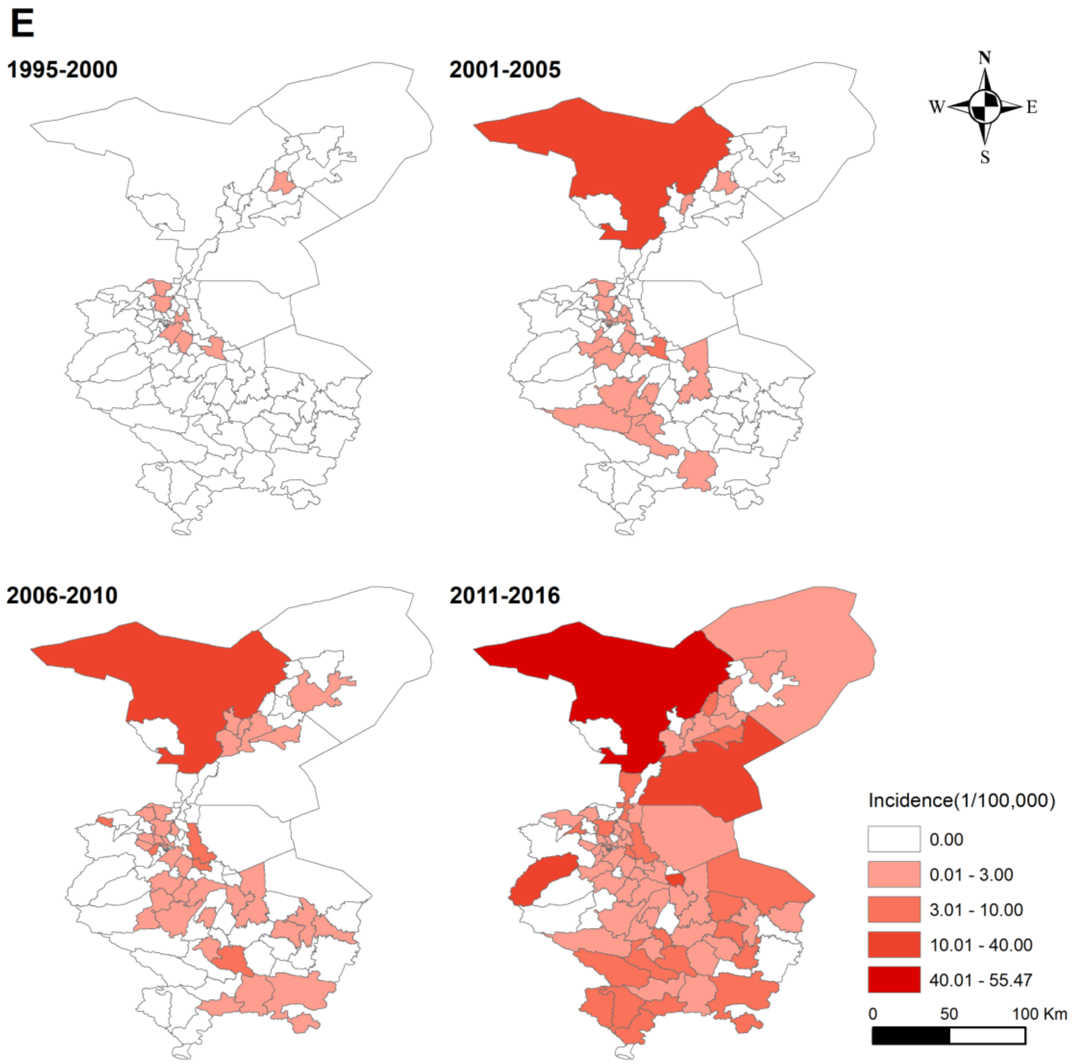
E
1995-2000
2001-2005
2006-2010
2011-2016
N
W
E
S
Incidence(1/100,000)
0.00
0.01 - 3.00
3.01 - 10.00
10.01 - 40.00
40.01 - 55.47
0
50
100 Km

Figure S3-F

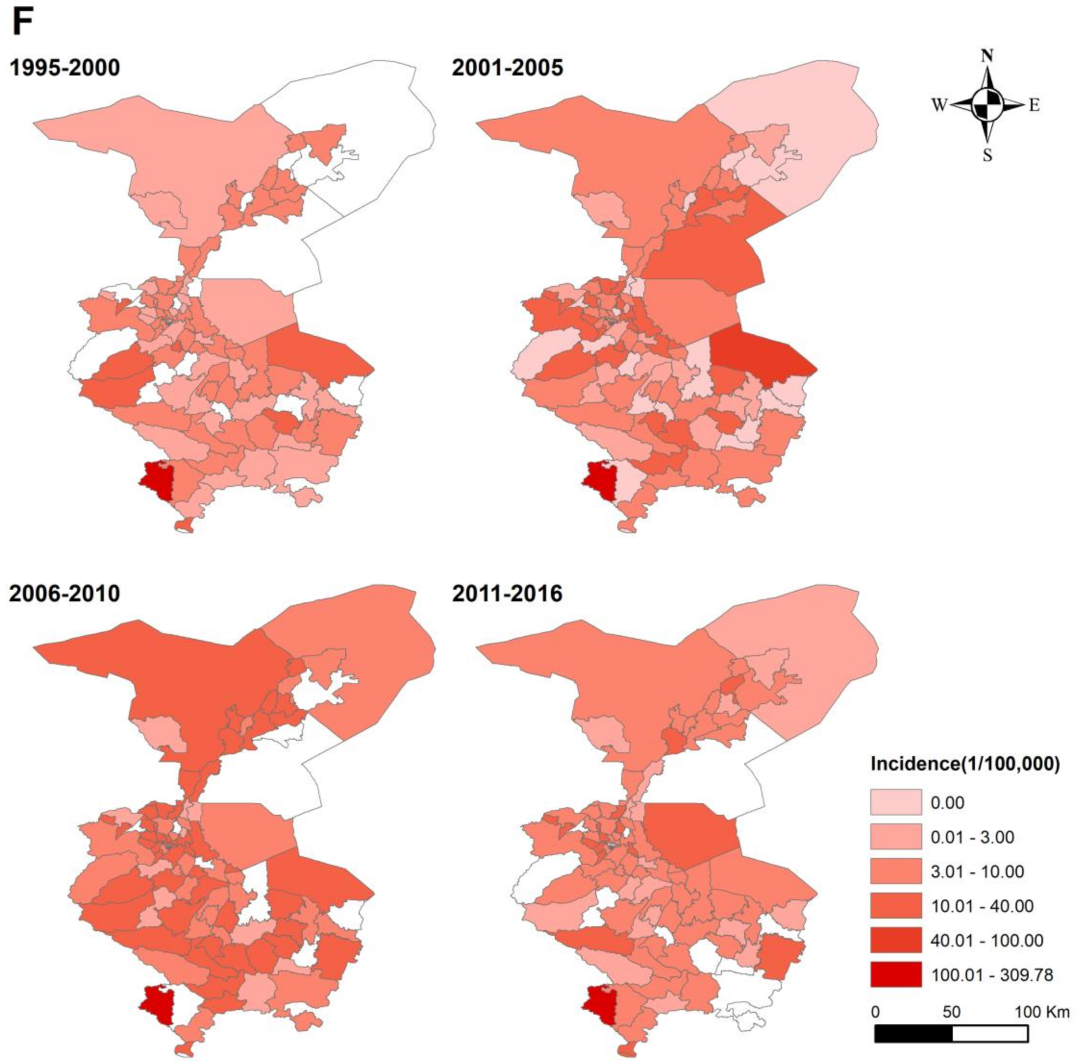
F
1995-2000
2001-2005
2006-2010
2011-2016
N
W
E
S
Incidence(1/100,000)
0.00
0.01 - 3.00
3.01 - 10.00
10.01 - 40.00
40.01 - 100.00
100.01 - 309.78
0
50
100 Km
